# An audit of a high caloric refeeding regimen used for medically unstable adolescent inpatients with severe restrictive eating disorders

**DOI:** 10.1186/2050-2974-2-S1-O20

**Published:** 2014-11-24

**Authors:** Danielle Hewitt, Gail Anderson, Andrew Kennedy, Linette Gomes, Elizabeth Parker, Christine Wearne, Michael Kohn, Simon Clarke

**Affiliations:** 1 Department of Adolescent Medicine, Westmead Hospital, Sydney, Australia; 2 Department of Dietetics and Nutrition, Westmead Hospital, Sydney, Australia; 3 Department of Medical Psychology, Westmead Hospital, Sydney, Australia

## Aim

To present data on outcomes including weight and BMI gain, length of stay and complications of a high caloric refeeding regimen.

## Methods

A retrospective audit of 98 consecutive admissions over 36 months of medically unstable patients (Defined as bradycardic HR ≤50), who had a BMI of ≤18.5 kg/m2. Patients were prescribed a regimen of nasogastric feeds and oral diet graded up from 2400 Kcal/day to over 4000 Kcal/day. Prophylactic oral phosphate was routinely prescribed.

## Results

Median age 16.6 years (range: 14.7 - 19.9), median BMI 16.2 kg/m2 (12.4 - 18.5) on admission. Median weight gain in first week 4.0 kg (1.2 – 6.9), median total weight gain 7.8 kg (3.3 - 18.3). Median BMI on discharge 19.1 kg/m2 (16.4 - 21.0). Median length of stay 24.4 days (6.0 – 82.3 days). No admissions resulted in refeeding syndrome. Peripheral oedema and/or mild electrolyte abnormality occurred in 20.4% of admissions.

## Conclusion

It is possible to use a refeeding regimen that is higher in total caloric intake than currently recommended approaches, including NICE guidelines. With specialist medical and nursing care, relatively rapid weight gain and clinical stabilisation occurs with minimal complications. A prospective comparison between centres or a randomised controlled trial would contribute further to the evidence in this field.

This abstract was presented in the **Service Initiatives: Child and Adolescent Refeeding and FBT** stream of the 2014 ANZAED Conference.

